# Prevalence and Determinants of Work-Related Injuries Among Healthcare Workers in Jeddah, Saudi Arabia

**DOI:** 10.7759/cureus.36679

**Published:** 2023-03-25

**Authors:** Duaa S Alameer, Ibrahim R Noor Elahi

**Affiliations:** 1 Preventive Medicine, Ministry of Health, Jeddah, SAU

**Keywords:** back injury, saudi arabia, jeddah, healthcare workers, work-related injuries

## Abstract

Background and objective

Work-related injuries (WRIs) are a major occupational health issue among healthcare workers (HCWs) worldwide. Unsafe work environments, including physical, chemical, and biological hazards, are significant contributing factors to WRIs. However, the prevalence of WRIs among HCWs in Jeddah, Saudi Arabia, and their associated risk factors remain largely unexplored. In light of this, this study aimed to investigate the prevalence of WRIs and associated risk factors among HCWs in Jeddah, Saudi Arabia.

Methods

This was an analytic cross-sectional study conducted at secondary hospitals of the Ministry of Health (MOH) in Jeddah by using a self-administered questionnaire to measure the prevalence of WRIs and their related factors. The Chi-squared test was used to compare variables. A p-value <0.05 was considered statistically significant.

Results

The study involved 387 participants, of whom 283 (73.1%) were female. Most of the participants (n=226, 58.4%) agreed that personal protective equipment (PPE) was always available at their hospitals. Approximately two-thirds (n=251, 64.9%) agreed that they always used PPE. The overall prevalence of WRIs was 52%, with back injuries (32.6%), eye/mouth splashes (20.4%), and needle stick injuries (19.9%) being the most common. Years of work experience (p=0.014), type of profession (p<0.001), training in safety management (p=0.028), working hours (p=0.0001), working shifts (p=0.001), PPE availability (p=0.010), and sharp container availability (p=0.030) were significantly associated with WRIs.

Conclusion

This study revealed a high prevalence of WRIs among HCWs in Jeddah, Saudi Arabia, with back injuries, eye/mouth splashes, and needle stick injuries being the most common types. The study also found that the injuries were significantly associated with the type of profession, experience, work hours, and shifts as well as the availability of safety management and equipment such as sharp containers and PPE.

## Introduction

Healthcare workers (HCWs) encompass direct care providers such as physicians, pharmacists, laboratory technicians, and nurses, as well as indirect providers like healthcare administrators [1]. Work-related injuries (WRIs), for the purpose of this study, refer to self-reported injuries from incidental incidents in the past six months, including needle sticks, splashes to the face, wounds, contact with contaminated substances in the eyes or mouth, falls, rashes on the skin, burns, back injuries, electrical shocks, mechanical injuries, and other types of injuries [2]. HCWs face various health hazards at work, including biological, chemical, physical, and psychological hazards, affecting up to 50% of them [1].

The most significant occupational hazards in hospitals are primarily associated with biological infections caused by blood-borne or body fluid pathogens that can transmit directly from patients or indirectly through body fluids, biopsies, and patient fomites [3,4]. In addition, the coronavirus disease 2019 (COVID-19) pandemic and influenza outbreaks have been identified as causing major occupational hazards for HCWs [4]. Chemical hazards are also common in healthcare facilities, where several chemical agents are used for infection control or treating patients (e.g., pharmaceuticals, sterilants, and germicidal agents) [5].

Musculoskeletal risks are another significant risk factor for WRIs. About half of all musculoskeletal injuries sustained during patient care are caused by heavy and bulky items that need to be lifted and transported [6]. Psychological hazards manifest as deteriorating relationships with colleagues, irritability, indecisiveness, poor performance, increased smoking, alcohol consumption, and drug abuse among workers [7]. As a result, there is an increased rate of absenteeism, customer complaints, decreased satisfaction and morale, lack of engagement at work and in teamwork, and frequent conflicts with others, leading to a deteriorating quality of services [7,8]. An unsafe work environment leads to attrition of the health workforce, impacting the quality of care and outcomes [4]. Therefore, HCWs' work environment should adhere to strict safety policies, procedures, and practices. Providing a safe work climate and implementing safety practices play an essential role in reducing WRIs [9-11].

Studies have indicated that training, sleep quality, work experience, and safety protocols are major determinants of WRIs [12-14]. Furthermore, a US study has shown that working conditions and ergonomic factors are associated with WRIs [15]. In Malaysia, it was found that the unit of operation, workspace, noise, workload, administrative control, and types of tools used were associated with WRIs [16].

There is a dearth of studies on WRIs among HCWs in Saudi Arabia [17-20]. Therefore, this study aims to assess WRIs and their impact on HCWs in Jeddah, Saudi Arabia. The study objectives are to determine the prevalence and types of WRIs, their determinants, and organizational factors.

## Materials and methods

We conducted an analytic cross-sectional study from September to December 2022 in the Department of Preventive Medicine, Ministry of Health (MOH), Jeddah, Saudi Arabia. This study involved HCWs aged 24-60 years including physicians, nurses, pharmacists, radiology staff, laboratory specialists, dental staff, and allied medical professionals. Internists, medical students, and pregnant HCWs were excluded from the study. According to 2020 statistics from the MoH in Jeddah, the total number of HCWs in Jeddah is 18,343. Of these, physicians accounted for 25% (female: 2,130, male: 2,462), while 40% (female: 5,787, male: 1,557) were nurses, 1.6% (female: 126, male: 161) were pharmacists, and 33.4% (female: 2,212, male: 3,908) were allied personnel [18]. Using Raosoft with a confidence level of 95% and a margin of error of 5%, a minimum sample size of 387 was calculated from a total number of 18,343 HCWs in Jeddah. A multistage sampling technique was used to select the participants. Data collection was done through a self-administered, validated questionnaire previously used in similar studies [2].

Section A of the questionnaire included questions about sociodemographic characteristics such as gender, age, occupational category, nationality, marital status, profession, years of experience, past medical history, previous safety training, type of hospital, availability of personal protective equipment (PPE), and WRIs. Section B contained questions about the prevalence and frequency of occupational hazards, including physical, chemical, psychosocial, and biological injuries. Section C comprised questions related to different types of occupational hazards, including biological, physical, chemical, and psychosocial injuries.

The study was approved by the IRB of the Research Committee of the Saudi Board for Preventive Medicine in Jeddah city and the Directorate of Health Affairs in MOH (Approval# A01415). Written informed consent was obtained from each HCW participant. To ensure data confidentiality, all information was kept confidential for the sole purpose of this study.

Data were entered and analyzed using IBM SPSS Statistics version 26 (IBM Corp., Armonk, NY). Descriptive statistics such as frequencies and percentages were calculated to summarize nominal and ordinal data, while means and standard deviations (SD) were used to describe numerical variables. The Chi-squared test was applied to evaluate the association between categorical determinants and the outcome variables. Logistic regression analysis was performed to identify significant predictors of WRIs. A p-value <0.05 was considered statistically significant.

## Results

A total of 387 participants were included in this study, with the majority being female (n=283, 73.1%). The most common age group was 31-35 years with 149 participants (38.5%). The majority of participants were Saudi nationals (n=372, 96.1%), with 223 (57.6%) having a bachelor's degree. More than 10 years of experience was reported by the majority of participants (n=145, 37.5%). Table 1 shows the detailed demographic characteristics of the cohort.

**Table 1:** Demographic characteristics of study participants (n=387)

Characteristics	Frequency (n)	Percentage (%)
Age group, years		
18–25	43	11.1
26–30	84	21.7
31–35	149	38.5
36–40	61	15.8
41–45	29	7.5
46–50	8	2.1
51–55	6	1.6
56–60	3	0.8
>60	4	1.0
Gender		
Male	104	26.9
Female	283	73.1
Marital status		
Single	148	38.2
Married	213	55.0
Divorced	25	6.5
Widowed	1	0.3
Nationality		
Saudi	372	96.1
Non-Saudi	15	3.9
Educational level		
Diploma	86	22.2
Bachelor's	223	57.6
Postgraduate	78	20.2
Residential area		
Urban	368	95.1
Semi-urban	18	4.7
Rural	1	0.3
Smoking history		
Never smoker	254	65.6
Previous smoker	35	9.0
Current smoker	98	25.3
Work experience, years		
<1	49	12.7
1–4	86	22.2
5–10	107	27.6
>10	145	37.5
Chronic disease	52	13
Profession		
Physician	52	13.4
Nurse	137	35.4
Pharmacist	67	17.3
Radiology staff	24	6.2
Laboratory staff	59	15.2
Dentist	16	4.1
Public health employee	20	5.1
Dental technician	12	3.1

A total of 155 participants (40.1%) had received training in safety management, while 177 (45.7%) had received training in worker safety. Regarding patient load, 179 participants (46.3%) reported seeing less than 50 patients per week, 110 (28.4%) saw between 51-100 patients, and 60 (15.5%) saw more than 200 patients. In terms of working hours, 235 (60.7%) worked eight hours per day, 126 (32.6%) worked 8-12 hours, and only 26 (6.7%) worked more than 12 hours.

In terms of the availability of PPE, the majority of participants (n=226, 58.4%) reported that PPE is always available in their workplace. Furthermore, 100 (25.8%) participants stated that it is usually available, while 42 (10.9%) reported that it is sometimes available. A smaller number of participants reported that PPE is rarely available (n=8, 2.1%) or never available (n=11, 2.8%). Regarding the availability of sharp containers, the majority of participants (n=295, 76.2%) indicated that they were always available in their workplace. Additionally, 37 (9.6%) reported that they were usually available, while 19 (4.9%) said that they were sometimes available. A smaller proportion of participants reported that sharp containers were rarely available (n=12, 3.1%) or never available (n=24, 6.2%).

Regarding the use of PPE, the majority of participants (n=251, 64.9%) reported that they always use PPE in their workplace. Additionally, 60 (15.5%) reported that they usually use it, while 26 (6.7%) reported that they use it sometimes. A smaller proportion of participants reported that they use PPE rarely (n=19, 4.9%) or never (n=31, 8%).

The results of our study revealed that the overall incidence rate of WRIs was 52%. Among the reported injuries, back injuries were the most frequent, accounting for 32.6% of all WRIs. Eye/mouth splash injuries were the second most common type of injury at 20.4%, followed by needle stick injuries at 19.9%. Sharp injuries accounted for 17.6% of WRIs and fall-related injuries accounted for 12.4%. Direct contact with contaminated materials was responsible for 9% of all WRIs, while skin rashes accounted for 8%. Poison injuries accounted for 3.9% of WRIs, burn injuries accounted for 2.1%, and electrical shock accounted for 2.1%.

A Chi-square test was utilized to compare WRIs with demographic data. Results revealed that only years of work experience (p=0.014) and profession (p<0.001) displayed a significant correlation with WRIs (Table 2).

**Table 2:** Association between work-related injuries and demographic data

Demographic data	Occupational hazard		P-value
	Yes	No	
Age group, years			
18–25	22	21	0.324
26–30	51	33	
31–35	79	70	
36–40	23	38	
41–45	18	11	
46–50	4	4	
51–55	3	3	
56–60	1	2	
>60	2	2	
Gender			
Male	49	55	0.123
Female	154	129	
Marital status			
Single	79	69	0.747
Married	109	104	
Divorced	14	11	
Widowed	1	0	
Nationality			
Saudi	194	178	0.372
Non-Saudi	09	06	
Educational level			
Diploma	52	34	0.133
Bachelor's	116	107	
Postgraduate	35	43	
Residential area			
Urban	190	178	0.134
Semi-urban	13	05	
Rural	0	01	
Years of work experience			
<1	24	25	0.014
1–4	50	36	
5–10	43	64	
>10	86	59	
Profession			
Physician	18	34	<0.001
Nurse	93	44	
Pharmacist	25	42	
Radiology staff	10	14	
Laboratory staff	24	35	
Dentist	11	5	
Others	11	9	
Dental technician	11	1	

Similarly, the association of organizational factors with WRIs was analyzed, and the results revealed that participants who had received training in safety management had a significantly lower risk of WRIs (p=0.028). Moreover, working hours (p=0.0001), working shifts (p=0.001), availability of PPE (p=0.010), and sharp container availability (p=0.030) also showed a significant correlation with WRIs, as presented in Table 3.

**Table 3:** Association between organizational factors and work-related injuries

Factors	Occupational hazard		P-value
	Yes	No	
Trained in safety management	91	64	0.028
Trained in worker safety	99	78	0.124
Patients per week			
<50	92	87	0.910
51–100	60	50	
101–200	21	17	
>200	30	30	
Hours of working			
8	103	132	<0.001
8–12	82	44	
>12	18	08	
Work shift	93	132	
Morning, evening	09	13	<0.001
Mixed	101	39	
Health facility			
Hospital	191	173	0.827
PHC	12	11	
Personal protective equipment availability			
Never	4	7	0.010
Rarely	8	0	
Sometimes	22	20	
Usually	61	39	
Always	108	118	
Availability of sharp container			
Never	6	18	0.030
Rarely	8	4	
Sometimes	9	10	
Usually	24	13	
Always	156	139	
Use of personal protective equipment			
Never	11	20	0.191
Rarely	8	11	
Sometimes	16	10	
Usually	35	25	
Always	133	118	

The analysis revealed that participants with a lower level of education had higher odds of experiencing occupational hazards compared to those with higher education levels (p=0.001). Moreover, those with lower work experience had 1.349 times higher odds of experiencing occupational hazards compared to those with higher work experience (p=0.037). The results of the multinomial logistic regression analysis examining the association between occupational hazards and demographic data are shown in Table 4.

**Table 4:** Multinominal logistic regression analysis of the association between demographic data and occupational hazard

Occupational hazard (yes)	Adjusted odds ratio	95% confidence interval		P-value
		Lower bound	Upper bound	
Age	0.808	0.651	1.002	0.052
Gender	1.319	0.825	2.111	0.248
Marital status	1.068	0.735	1.551	0.730
Nationality	2.066	0.679	6.284	0.201
Educational level	3.780	1.561	5.085	0.001
Residential area	1.918	0.746	4.931	0.176
Years of work experience	1.349	1.018	1.787	0.037
Profession	1.045	0.944	1.157	0.400

The results showed that participants who saw more patients per week had 2.4 times higher odds of experiencing occupational hazards compared to those who saw fewer patients (p=0.044). Similarly, participants working the night shift had 1.7 times higher odds of experiencing occupational hazards compared to those working the morning shift (p<0.001) (Table 5).

**Table 5:** Multinominal logistic regression analysis of the association between organizational factors and occupational hazard

Occupational hazard (yes)	Adjusted odds ratio	95% confidence interval		P-value
		Lower bound	Upper bound	
Trained in safety management	1.242	0.665	2.322	0.496
Training in worker safety	1.043	0.857	1.270	0.675
Patients per week	2.480	1.011	3.167	0.044
Work shift	1.774	1.382	2.277	0.000
What is the type of health facility where you work now?	1.173	0.587	2.346	0.652
How is the availability of personal protective equipment in the hospital?	0.793	0.602	1.045	0.099
How is the availability of sharp object containers in the hospital?	1.164	0.926	1.464	0.192
How frequently do you use personal protective equipment?	1.145	0.935	1.403	0.191

## Discussion

This study revealed that the prevalence of WRIs was 52%, which is higher than the 36.5% reported in a previous study conducted in Ethiopia [21]. A study carried out in Turkey also found a high injury rate among nurses, with medication administration being the most common cause of injury [22]. In contrast, a study conducted in Ghana reported a lower prevalence (39.7%), with needlesticks and sharp objects being the primary causes of injury. The findings of our study indicate that back injuries were the most common type of injury, reported by approximately one-third of the participants, followed by eye/mouth splash injuries and needlestick injuries [23].

A study conducted in Singapore reported an overall incidence of sharp injuries and splash exposure of 28.9 per 1,000 health workers, with the highest incidence among doctors (43.7%) and nurses (37.7%) [24]. This study also revealed that WRIs were associated with the type of profession and work experience, which may influence the type of injuries sustained. Our study included mostly nurses, which could explain the high rates of back injuries reported, consistent with previous studies that have shown that at least three-quarters of nurses experience back pain at some point in their work [19,25]. The nature of the work of nurses, which involves bending and twisting, lifting and pulling objects, and manually handling patients, maybe the leading cause of low back pain [19]. In another study, nurses with higher education levels, specifically those with master's and doctoral degrees, those employed in internal medicine and pediatric ICUs, and those working in shifts, reported higher rates of back pain [25], likely due to the demanding nature of their work.

A study that assessed the prevalence and risk factors of needlestick injuries reported an incidence rate of at least one event per year of 22.2, with 53.8% of injuries going unreported, and physicians, nurses, and dentists being the most susceptible [18]. While Abalkhail et al. [18] found that HCWs aged 26-30 years were 2.5 times more likely to experience needle injuries than other age groups, our study did not find any statistically significant differences in WRIs based on age. However, our study results are consistent with Abalkhail et al. [18] in finding that the type of work/profession, such as handling needles, and work experience were associated with injuries.

In line with a previous study conducted in Ethiopia [21], our study also found that the absence of PPE and sharp containers, working for more than eight hours per day, and working the night shift were associated with higher risks of WRIs. The absence of PPE is expected to result in injuries, while long working hours and night shifts can lead to fatigue, which causes reduced attention and subsequent injuries [8]. This is supported by a study conducted in Australia, which showed that fatigue and workplace stress were predictors of near misses, and safety control led to reduced incidences. The study also found that fatigue increases the risks of WRIs [10].

It has been suggested that paying attention to safety protocols and providing organizational training to improve the safety-related behaviors of the workforce can decrease WRIs [10]. Our study supports this, as it found a significant association between WRIs and the lack of PPE as well as the lack of sharp containers. Research has indicated that there is a correlation between safety performance and occupational accidents and injuries, as well as safety climate and employees' safety performance [26]. This aligns with our finding that participants trained in safety management were significantly at a lower risk of WRIs. Moreover, studies evaluating the impact of overtime and long work hours on occupational injuries revealed that rates of injuries increase proportionally with the increase in work hours per week [27]. In a recent study conducted to assess the association between working hours and injuries in hospital shift work, the authors found a higher risk of WRIs during evening shifts and the workdays that follow night shifts, with the risk rising with the frequency of evening shifts but not night shifts [28].

This study has some limitations, including its cross-sectional design, which could not identify a causal effect. Additionally, the study was conducted using questionnaires, which are prone to recall bias and subjective observations, which might affect the generalization of results.

## Conclusions

This study revealed a high prevalence of WRIs among HCWs in Jeddah, Saudi Arabia, with back injuries, eye/mouth splash injuries, and needle stick injuries being the most common types. We also found a correlation between injuries and the type of profession and experience, work hours, shifts as well as the availability of safety management and equipment, such as sharp containers and PPE. These findings underscore the need for hospitals in Jeddah to revise their safety protocols and establish safety awareness campaigns aimed at identifying risk factors and improving working conditions to protect the workforce and enhance the quality of care. More research, preferably extensive longitudinal and prospective studies, is recommended to further explore the impact of WRIs on healthcare services and outcomes.

## Appendices

**Table 6:** Questionaire used in the study (Prevalence and Determinants of Work-Related Injuries Among Healthcare Workers in Jeddah, Saudi Arabia)

Section 1: Personal information	Response	
Age		
Gender	Male	Female
Marital status	Single	Married
	Divorced	Widowed
Nationality	Saudi	Non-Saudi
Years of experience	<1 year	1-4 years
	5-10 years	>10 years
Profession	Physician	Nurse
	Pharmacist	Radiology staff
	Laboratory staff	Dental staff
	Other	
Past medical history	Yes	No
What is the type of the hospital?	General hospital	Specialized hospital
Did you have training in management safety?	Yes	No
Did you have training in worker safety?	Yes	No
How is the availability of personal protective equipment in the hospital?	Never/sometimes/always	Rarely/usually
How is the availability of sharp object containers in the hospital?	Never/sometimes/always	Rarely/usually
How frequently do you use personal protective equipment?	Never/sometimes/always	Rarely/usually
Section 2: Work-related injuries		
Have you experienced needle-stick injuries at your workplace in the last six months?	Never/1 time/2 times/3 times	4 times/5 times/6 and more
Have you experienced injuries from contaminated objects at your workplace in the last six months?	Never/1 time/2 times/3 times	4 times/5 times/6 and more
Have you experienced back injuries at your workplace in the last six months?	Never/1 time/2 times/3 times	4 times/5 times/6 and more
Have you experienced eye/mouth splashes at your workplace in the last six months?	Never/1 time/2 times/3 times	4 times/5 times/6 and more
Have you experienced cuts with sharp objects at your workplace in the last six months?	Never/1 time/2 times/3 times	4 times/5 times/6 and more
Have you experienced skin rashes related to your work in the last six months?	Never/1 time/2 times/3 times	4 times/5 times/6 and more
Have you experienced injuries due to falls at your workplace in the last six months?	Never/1 time/2 times/3 times	4 times/5 times/6 and more
Have you experienced burn injuries at your workplace in the last six months?	Never/1 time/2 times/3 times	4 times/5 times/6 and more
Have you experienced poisoning symptoms related to your workplace in the last six months?	Never/1 time/2 times/3 times	4 times/5 times/6 and more
Have you experienced electrical shocks at your workplace in the last six months?	Never/1 time/2 times/3 times	4 times/5 times/6 and more
Section 3: Occupational hazards:		
Do you have direct contact with patients?	Never/rarely	Sometimes/often/always
Do you have direct contact with chemical products?	Never/rarely	Sometimes/often/always
Do you have direct contact with radiation?	Never/rarely	Sometimes/often/always
Do you have direct contact with the patient blood and body fluids or tissues?	Never/rarely	Sometimes/often/always
Do you have direct contact with contaminated sharp objects?	Never/rarely	Sometimes/often/always
Do you work in an environment where there is high temperature variation (highly cold or hot temperature)?	Never/rarely	Sometimes/often/always
Do you work in an environment where there is loud noise?	Never/rarely	Sometimes/often/always
Do you work in an environment where there is a lack of space?	Never/rarely	Sometimes/often/always
Do you work in an environment where there is poor air quality with little or no ventilation?	Never/rarely	Sometimes/often/always
Do you work in an environment where there is poor lighting?	Never/rarely	Sometimes/often/always
Do you work in an environment where there is a risk of falls or trips?	Never/rarely	Sometimes/often/always
Do you work in an environment where there are electrical hazards?	Never/rarely	Sometimes/often/always

## References

[1] Rafique R. Sehar S, Afzal MN (2019). Health hazards at work place: application of WHO modal with literature review. Saudi J Nurs Health Care.

[2] Gimeno D, Felknor S, Burau KD, Delclos GL (2005). Organisational and occupational risk factors associated with work related injuries among public hospital employees in Costa Rica. Occup Environ Med.

[3] Rim KT, Lim CH (2014). Biologically hazardous agents at work and efforts to protect workers' health: a review of recent reports. Saf Health Work.

[4] Ghosh T (2013). Occupational health and hazards among health care workers. Int J Occup Saf Health.

[5] McDiarmid MA (2006). Chemical hazards in health care: high hazard, high risk, but low protection. Ann N Y Acad Sci.

[6] Ribeiro T, Serranheira F, Loureiro H (2017). Work related musculoskeletal disorders in primary health care nurses. Appl Nurs Res.

[7] Okeafor CU, Alamina FE (2018). A qualitative study on psychosocial hazards among health care workers in a tertiary health facility in south-south Nigeria. Ann Ib Postgrad Med.

[8] Mbombi MO, Mothiba TM, Malema RN, Malatji M (2018). The effects of absenteeism on nurses remaining on duty at a tertiary hospital of Limpopo province. Curationis.

[9] Huang YH, Ho M, Smith GS, Chen PY (2006). Safety climate and self-reported injury: assessing the mediating role of employee safety control. Accid Anal Prev.

[10] Strahan C, Watson B, Lennonb A (2008). Can organisational safety climate and occupational stress predict work-related driver fatigue?. Transp Res Part F: Traffic Psychol Behav.

[11] Ali H, Azimah CN, Subramaniam C (2009). Management practice in safety culture and its influence on workplace injury: an industrial study in Malaysia. Disaster Prev Manag.

[12] Strid EN, Wåhlin C, Ros A, Kvarnström S (2021). Health care workers' experiences of workplace incidents that posed a risk of patient and worker injury: a critical incident technique analysis. BMC Health Serv Res.

[13] Maske UE, Riedel-Heller SG, Seiffert I, Jacobi F, Hapke U (2016). Prevalence and comorbidity of self-reported diagnosis of burnout syndrome in the general population - results of the German Health Interview and Examination Survey for Adults (DEGS1). Psychiatr Prax.

[14] Aderaw Z, Engdaw D, Tadesse T (2011). Determinants of occupational injury: a case control study among textile factory workers in Amhara Regional State, Ethiopia. J Trop Med.

[15] Hamadi H, Probst JC, Khan MM, Bellinger J, Porter C (2018). Determinants of occupational injury for US home health aides reporting one or more work-related injuries. Inj Prev.

[16] Obi A, Ariffin A, Zainuddin H (2017). Factors associated with work related injuries among workers of an industry in Malaysia. Int J Public Health Clin Sci.

[17] Al-Dawood KM (2000). Non-fatal occupational injuries admitted to hospitals among general organization for social insurance workers in Al-Khobar city, Saudi Arabia: experience of one year. J Family Community Med.

[18] Abalkhail A, Kabir R, Elmosaad YM (2022). Needle-stick and sharp injuries among hospital healthcare workers in Saudi Arabia: a cross-sectional survey. Int J Environ Res Public Health.

[19] Al Amer HS (2020). Low back pain prevalence and risk factors among health workers in Saudi Arabia: a systematic review and meta-analysis. J Occup Health.

[20] Rezaei B, Mousavi E, Heshmati B, Asadi S (2021). Low back pain and its related risk factors in health care providers at hospitals: a systematic review. Ann Med Surg (Lond).

[21] Ayenew E, Akafu W, Wolde Daka D (2022). Prevalence of work-related health hazard and associated factors among health workers in public health institutions of Gambella Town, Western Ethiopia: cross-sectional survey. J Environ Public Health.

[22] Erturk Sengel B, Tukenmez Tigen E, Bilgin H, Dogru A, Korten V (2021). Occupation-related injuries among healthcare workers: incidence, risk groups, and the effect of training. Cureus.

[23] Appiagyei H, Nakua EK, Donkor P, Mock C (2021). Occupational injuries among health care workers at a public hospital in Ghana. Pan Afr Med J.

[24] Leong XY, Yee FZ, Leong YY, Tan SG, Amin IB, Ling ML, Tay SM (2019). Incidence and analysis of sharps injuries and splash exposures in a tertiary hospital in Southeast Asia: a ten-year review. Singapore Med J.

[25] Ovayolu O, Ovayolu N, Genc M, Col-Araz N (2014). Frequency and severity of low back pain in nurses working in intensive care units and influential factors. Pak J Med Sci.

[26] Clarke S (2006). The relationship between safety climate and safety performance: a meta-analytic review. J Occup Health Psychol.

[27] Dembe AE, Erickson JB, Delbos RG, Banks SM (2005). The impact of overtime and long work hours on occupational injuries and illnesses: new evidence from the United States. Occup Environ Med.

[28] Härmä M, Koskinen A, Sallinen M, Kubo T, Ropponen A, Lombardi DA (2020). Characteristics of working hours and the risk of occupational injuries among hospital employees: a case-crossover study. Scand J Work Environ Health.

